# Environmental Impacts of Pig and Poultry Production: Insights From a Systematic Review

**DOI:** 10.3389/fvets.2021.750733

**Published:** 2021-10-27

**Authors:** Ines Andretta, Felipe M. W. Hickmann, Aline Remus, Carolina H. Franceschi, Alexandre B. Mariani, Catiane Orso, Marcos Kipper, Marie-Pierre Létourneau-Montminy, Candido Pomar

**Affiliations:** ^1^Department of Animal Science, Universidade Federal do Rio Grande do Sul, Porto Alegre, Brazil; ^2^Département des Sciences Animales, Faculté des Sciences de l'Agriculture et de l'Alimentation, Université Laval, Québec, QC, Canada; ^3^Sherbrooke Research and Development Centre, Agriculture and Agri-Food Canada, Sherbrooke, QC, Canada; ^4^Elanco Animal Health, São Paulo, Brazil

**Keywords:** sustainability, swine, broilers, environment, livestock, climate change, laying hens, precision feeding

## Abstract

Pig and poultry production systems have reached high-performance levels over the last few decades. However, there is still room for improvement when it comes to their environmental sustainability. This issue is even more relevant due to the growing demand for food demand since this surplus food production needs to be met at an affordable cost with minimum impact on the environment. This study presents a systematic review of peer-reviewed manuscripts that investigated the environmental impacts associated with pig and poultry production. For this purpose, independent reviews were performed and two databases were constructed, one for each production system. Previous studies published in peer-reviewed journals were considered for the databases if the method of life cycle assessment (LCA) was applied to pig (pork meat) or poultry (broiler meat or table eggs) production to estimate at least the potential effects of climate change, measured as CO_2_-eq. Studies considering the cradle-to-farm gate were considered, as well as those evaluating processes up to the slaughterhouse or processor gate. The pig database comprised 55 studies, while 30 publications were selected for the poultry database. These studies confirmed feeding (which includes the crop cultivation phase, manufacturing processes, and transportation) as the main contributor to the environmental impact associated with pig and poultry production systems. Several studies evaluated feeding strategies, which were indicated as viable alternatives to mitigate the environmental footprint associated with both production chains. In this study, precision feeding techniques are highlighted given their applicability to modern pig and poultry farming. These novel feeding strategies are good examples of innovative strategies needed to break paradigms, improve resource-use efficiency, and effectively move the current productive scenario toward more sustainable livestock systems.

## Introduction

The increasing demand for food is an important challenge that society will face in the coming decades. The growing population will need more resources, leading to a relevant increase in food demand. The productive sector (including agriculture and livestock) needs to support the growing demands for food, however, without compromising the ability of the future generations to also meet their requirements. In other words, environmentally sustainable agri-food systems are mandatory requirements for a world with increasing urbanization and growing food demands.

In this context, the benefits of agri-food sectors for society need to be maximized (1), which can be achieved by improving the efficiency in which the resources are applied in the production chains. The current production methods will need to adapt to these new challenges (limited resources, increased production), with most surplus food production being supplied by innovative agri-food systems (2).

Pig and poultry production systems have reached high-performance levels over the last few decades. Together, these sectors provide a large amount of affordable and nutritious food, especially high-quality protein, contributing to food security worldwide. However, there is still room for improvement when it comes to their environmental sustainability. Feeding pigs and poultry requires tremendous amounts of feed resources, with several studies indicating it as an important source of environmental impact (3). In addition, pigs and broilers excrete annually large amounts of nitrogen and phosphorus to the environment, which conditions the production sustainability of these chains (4).

Conventionally, the impacts of pig and poultry production have been assessed by methodologies that used an “animal basis” approach (e.g., studies focusing on reducing nutrient excretion). These are very important studies; however, few mitigation strategies have focused on the efficiency of resource use, which is critical in a global context. Considering the relevance of the topic, it is important to investigate feeding practices that mitigate the environmental impacts associated with the entire production system. Thus, we carried out a systematic review to summarize, analyze, and compare studies that used life cycle assessment (LCA) to evaluate the environmental impacts associated with pig and poultry production systems.

## Materials and Methods

This systematic review was based on structured and elaborated research performed using online search methods. The search strategy was planned and carried out to identify as many studies as possible on the subject. Papers were rigorously selected and those focusing on feeding practices were further evaluated.

Independent searches were performed for pig and poultry production systems. The strategy “PICo” was applied to build the research question by identifying “Population” (database 1: “pig”; database 2: “poultry”), “Interest” (“life cycle assessment”), and “Context” (“climate change”) for both searches. Alternative terms for population and interest were listed using synonymous words in English to compose the final search strategy. Context was applied later (through full-text reads) to avoid missing any study in which the response was not mentioned among the main terms (title, abstract, and keywords). The final search terms were:

Database 1:

(*pig OR pigs OR swine*) *AND* (“*life cycle assessment*” *OR* “*life cycle*” *OR* “*carbon emission*” *OR* “*carbon footprint*” *OR* “greenhouse gas^*^” *OR* “*global warming*” *OR LCA*)

Database 2:

(poultry OR broiler^*^ OR chicken^*^OR hen) *AND* (“*life cycle assessment*” *OR* “*life cycle*” *OR* “*carbon emission*” *OR* “*carbon footprint*” *OR* “greenhouse gas^*^” *OR* “*global warming*” *OR LCA*).

The search was conducted in March 2020, considering only original peer-reviewed studies published in scientific journals available in PubMed, Scopus, and Web of Science. A snowball approach using forward (e.g., databases) and backward research methods (e.g., direct journal search, reference lists, studies listed in previously published reviews) was performed to increase the chance of including as many relevant studies as possible. No limitations on the geographic origin or year of publication were applied in both searches.

Each database was exported to the reference software (EndNote X9, Philadelphia, PA) used to organize references and manage part of the study selection. Duplicate references were identified and excluded. Studies were critically evaluated regarding their relevance and quality by examining titles and abstracts, followed by a complete review of the LCA study. Two reviewers performed a critical evaluation of the study eligibility. A study was not considered in the final database (removed) after mutual agreement, with a third reviewer reassessing studies that differed in terms of eligibility.

The selection criteria were stated as (i) original papers published in peer-reviewed journals, (ii) environmental impact evaluated using the LCA methodology; (iii) evaluation of pig (pork meat) or poultry (broiler meat or table eggs) production systems; (iv) scopes including cradle-to-farm, to the slaughterhouse, or to processor gate; (v) estimation of at least the potential impact of climate change, in CO_2_-eq. The quality of selected studies was further evaluated and information relevant to describe the proposed theoretical model was transferred to the pig and poultry spreadsheets. Finally, cross-study comparisons were performed considering the subject, scope, and main results observed.

## Results

### Studies Focusing on Pig Production

**Figure 1:**
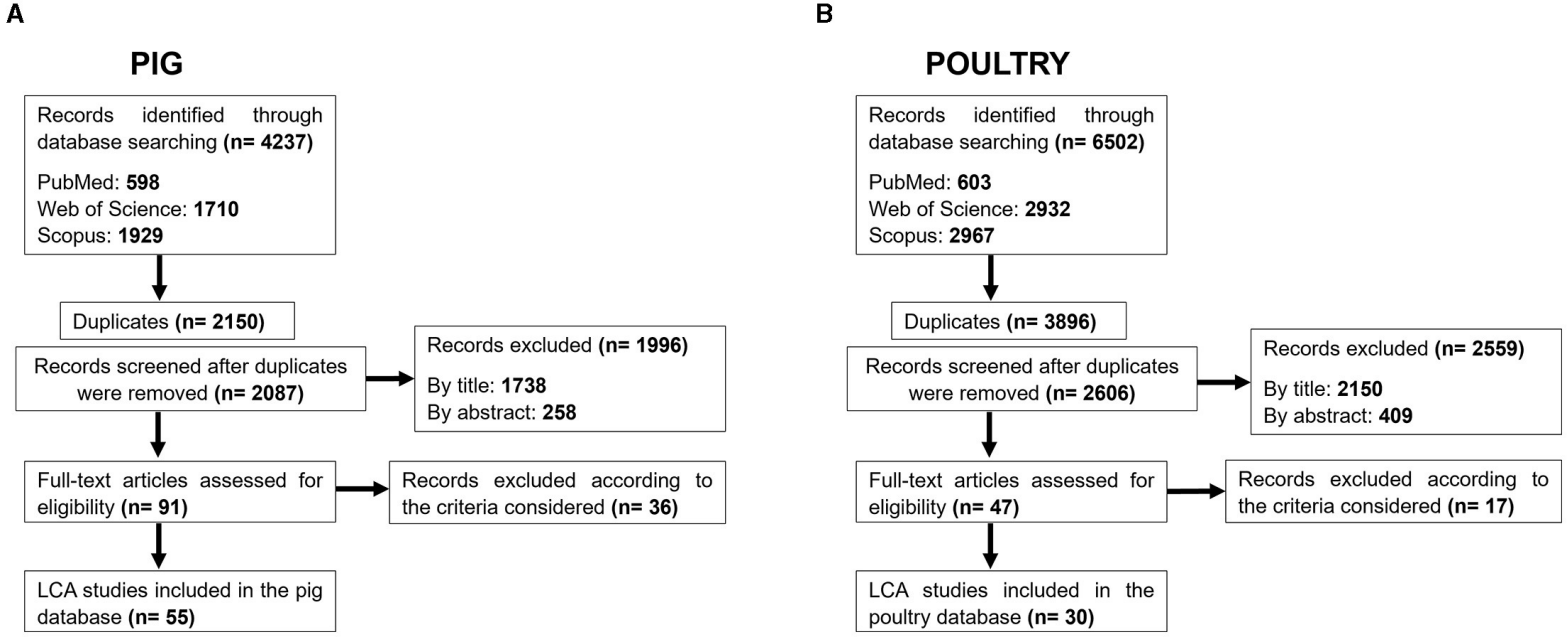
Study selection diagram for pig **(A)** or poultry **(B)** databases.

The research process until obtaining the final pig database is described in Figure 1A. Articles obtained by online searches (4,237 references) were critically evaluated and successive exclusions were performed. The main exclusions (more related to methodological aspects of the original studies) were performed when assessing the full-text, when 36 references were eliminated (criterium i and ii: 15 publications; criterium iii: 2 publications; criterium iv: 13 publications; and criterium v: 6 publications). The final list of 55 selected studies is described in Table 1.

**Table 1:** Summary of the LCA studies on pig production in terms of location, functional unit, and climate change potential.

**Code**	**Study**	**Country**	**Functional unit**	**Climate change potential^a^, CO_2_-eq**
1	Basset-Mens and van der Werf (5)	France	1 kg of live weight	2.30–3.97 kg
2	Eriksson et al. (6)	Sweden	1 kg of live weight gain	1.36–1.51 kg
3	Basset-Mens et al. (7)	France	1 kg of live weight	2.30 kg
4	Basset-Mens et al. (8)	France	1 t of pig	0.88–1.39 t
5	Liang et al. (9)	Japan	1 kg of carcass weight	5.02 kg
6	Halberg et al. (10)	Denmark	1 kg of live weight	2.80–3.30 kg
7	Halberg et al. (10)	United States	1 t live weight pig	2.47–3.33 kg
8	Aramyan et al. (11)	Europe, several countries	1 kg of slaughter weight	2.55–2.97 kg
9	Bonesmo et al. (12)	Norway	1 kg of carcass weight	2.65 kg
10	Devers et al. (13)	United Kingdom	1 kg of cut pork	2.55–4.5 kg
11	Dolman et al. (14)	Netherlands	100 kg of live weight	473–637 kg
12	Stone et al. (15)	United States	1 pig (118kg)	398.20 kg
13	De Moraes et al. (16)	World, several countries	1 kg of live weight pig	5.36–5.57 kg
14	Luo et al. (17)	China	1 farm (1,956 units of 500 kg each)	5,611–5,714 t
15	Ogino et al. (18)	Japan	1 kg of meat after dressing	7.12–7.12 kg
16	Reckmann et al. (19)	Germany	1 market pig	346–370 kg
17	Dourmad et al. (20)	Europe, several countries	1 kg of slaughter weight	3.20–3.25 kg
18	Jacobsen et al. (21)	Belgium	1 kg of live weight pig	2.25–3.47 kg
19	Sasu-Boakye et al. (22)	Sweden	1 kg of deboned pork	5.70 kg
20	Cherubini et al. (23)	Brazil	1 kg carcass weight	2.10–2.20 kg
21	Cherubini et al. (24)	Brazil	1 t of swine carcass	3.11–3.55 t
22	González-García et al. (25)	Portugal	30 kg of weight gain (finishing phase)	67.15–76.02 kg
23	Mackenzie et al. (26)	Canada	1 kg of meat (carcass weight)	3.34 kg
24	Reckmann and Krieter (27)	Germany	1 kg of carcass weight	2.81 kg
25	van Zanten et al. (28)	Netherlands	1 kg of slaughter weight	3.09–3.36 kg
26	Wang et al. (29)	China	1 kg of live weight pig	2.50 kg
27	Groen et al. (30)	Netherlands	1,000 pigs	9.08E+04 kg
28	Kebreab et al. (31)	Europe, North, and South America	1 kg of live weight	2.61 kg
29	Lamnatou et al. (32)	Spain	1 t of live weight pig	1.98–2.46 t
30	Mackenzie et al. (33)	Canada	1 kg of meat (live or carcass weight)	3.2–5.5 kg
31	Monteiro et al. (34)	Brazil and France	1 market pig (105 kg)	336–460 kg
32	Noya et al. (35)	Spain	1 kg of carcass weight	1.95–2.55 kg
33	Pirlo et al. (36)	Italy	1 kg of weight gain (fattening phase)	2.27–3.00 kg
34	Sagastume Gutiérrez et al. (37)	Cuba	1 kg of live-weight pig	6.70 kg
35	Wang et al. (38)	China	1 kg of carcass pork	8.70 kg
36	Ali et al. (39)	Brazil	1 kg of cut pork	10.3 kg
37	Bava et al. (40)	Italy	1 kg of live weight gain	3.3 kg
38	Li et al. (41)	China	1 pig (120kg)	1,019 kg
39	Monteiro et al. (42)	Brazil	1 market pig	2.29–3.19 kg
40	Noya et al. (43)	Spain	1 kg of live weight pig	1.13–1.96 kg
41	Noya et al. (44)	Spain	1 kg of live weight pig	2.69–5.81kg
42	Six et al. (45)	Belgium	1 market pig	248.53 kg
43	Andretta et al. (46)	Brazil	1 kg of weight gain (fattening phase)	2.57–2.67 kg
44	Rudolph et al. (47)	Europe, several countries	1 kg of cut pork	4.96 kg
45	Arrieta and González (48)	Argentina	100 kg live weight pig	342 kg
46	Monteiro et al. (49)	Brazil	100 g of pork	0.46 kg
47	Monteiro et al. (50)	Europe, several countries	1 t live weight pig	1.78–2.36 t
48	Ottosen et al. (51)	Denmark	1 t live weight pig	1.47–2.71 t
49	Reyes et al. (52)	Cuba	1 t of live weight pig	0.89–0.94 t
50	Anestis et al. (53)	Greece	1 kg of weight gain (nursery phase)	1.76–2.45 kg
51	Cadero et al. (54)	France	1 kg of live weight pig	5.07–9.35 kg
52	Garcia-Gudino et al. (55)	Spain	1 kg of live weight pig	4.18 kg
53	Horrillo and Gaspar (56)	Spain	1 kg of live weight pig	6.87–9.65 kg
54	Monteiro et al. (57)	Brazil	1 kg of live weight pig	3.85–4.15 kg
55	Pexas et al. (58)	Denmark	1 kg of live weight gain	2.16–2.48 kg

a *Original results were preserved, however, some conversions were needed for the purpose of having the same weight unit as the functional unit*.

**Figure 2:**
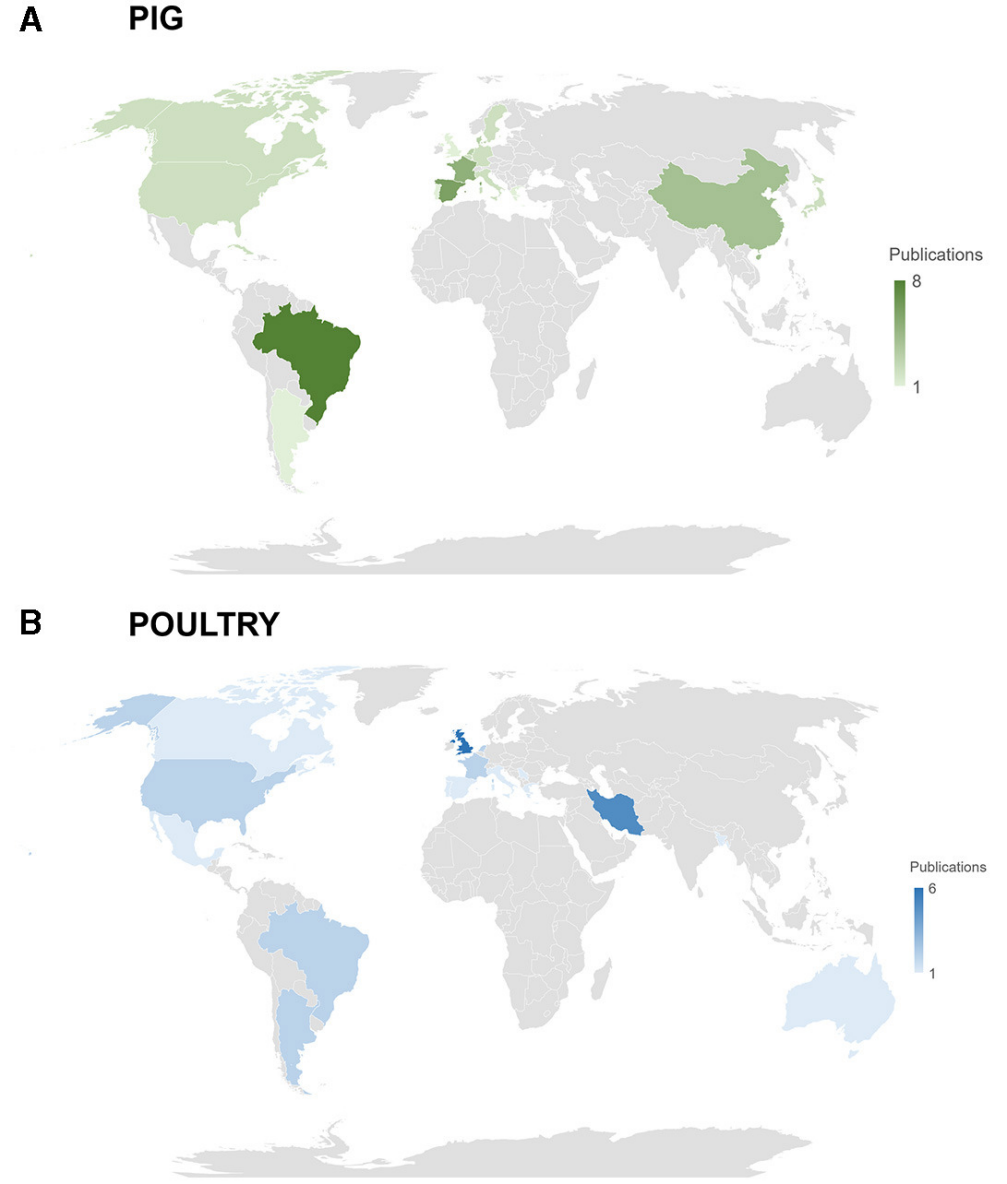
Location of the LCA studies focusing on pig **(A)** or poultry **(B)** production. Studies that simulated two or more countries, or even an entire continent, are not displayed in the figure.

The first LCA study identified in the pig database was published in 2005. Considering the entire database, 26 journals reported publications, with 16 papers being published in Journal of Cleaner Production and 5 papers in Animal. Production scenarios located in Brazil (which was considered in eight studies), Spain (considered in six studies), France (considered in five studies), and China (considered in four studies) were assessed in the selected papers, as illustrated in Figure 2A. The frequency of studied countries is highly related to the location of the main research groups. However, it is important to highlight that the order of most studied countries is not in complete agreement with the pork production ranking (led by China). Another important aspect related to the geographical characteristics of the papers is that five studies were developed by researchers from countries different than the one (or at least one of the regions) considered in the simulations, with Brazil or South America being studied in three of them.

A scope described as cradle-to-farm gate was used in the majority of the studies, which means that all phases comprised from the crop cultivation (and its inputs/outputs) up to the animal rearing phase were considered in these projects. The impacts associated with slaughtering and processing were considered in nine publications only.

**Figure 3:**
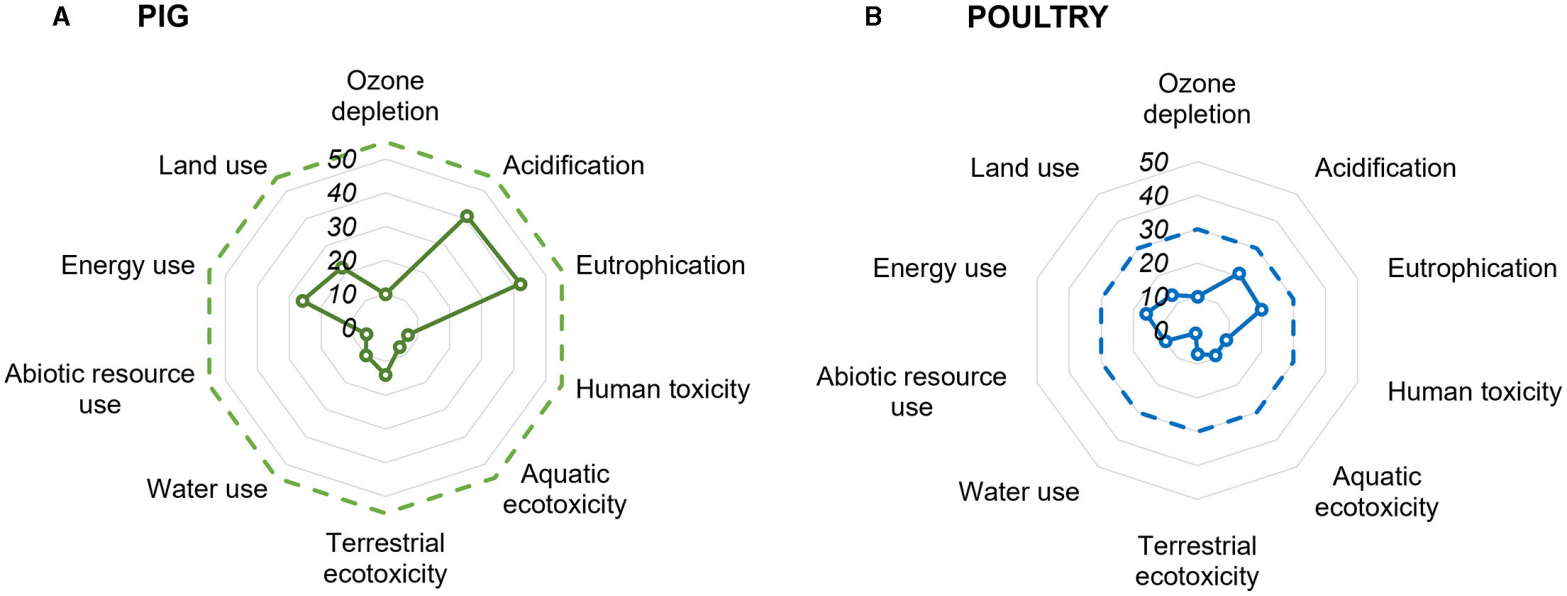
Environmental impact categories evaluated in the LCA studies focusing on pig **(A)** or poultry **(B)** production systems, with dashed lines indicating the total number of publications included in each database.

Climate change was the focus of our study. However, the LCA studies also reported other impact categories (Figure 3A). From those variables, the most prevalent were eutrophication and acidification, followed by the use of energy and land.

The main subjects under evaluation in the studies focusing on pig production are presented in Table 2. The characterization of pig production in the region or country was the main objective in 18 studies. Another important objective in the studies was the comparison of production systems (including organic or alternative housing systems), which was the main subject in nine papers.

**Table 2:** Summary of the LCA studies on pig production in terms of main subject under analysis and scope boundary.

**Code**	**Main study subject**	**Scope final boundary**
1	Production systems	At farm gate
2	Feed choice	At farm gate
3	Implications of uncertainty and variability	At farm gate
4	Production systems	At farm gate
5	Production in Japan	At farm gate
6	Production systems (organic)	At farm gate
7	Production systems	At farm gate
8	Production system in Europe	At farm gate
9	Production in Norway	At farm gate
10	Production in Western Cape and Flanders	Delivered to the distribution center
11	Production systems	At farm gate
12	Production in the United States	At farm gate
13	Immunological castration	At farm gate
14	Manure management	At farm gate
15	Low-protein diet supplemented with amino acids	At slaughterhouse gate
16	Production in Germany	At farm gate
17	Production systems	At slaughterhouse gate
18	Production in Flanders	At farm gate
19	Protein sources for feed production	At pork cutting gate
20	Manure management	At farm gate
21	Feed composition for finishing pigs	At slaughterhouse gate
22	Production in Portugal	At farm gate
23	Production in Canada	At the slaughterhouse gate
24	Farm performance	At farm gate
25	Replacing soybean meal with rapeseed meal	At slaughterhouse gate
26	Production in North China	At farm gate
27	Sensitivity analysis	At farm gate
28	Specialty feed ingredients	At farm gate
29	Production in Spain	At farm gate
30	Utilizing co-products as feed	At farm gate
31	Protein source, feeding programs (including precision feeding), amino acids inclusion	At farm gate
32	Production in Catalonia	At farm gate
33	Production in Italy (heavy pig)	At farm gate
34	Manure management	At farm gate
35	Husbandry on different scale	At slaughterhouse gate
36	Using co-products in the diets of finishing pigs	At slaughterhouse gate
37	Production system in Italy (heavy pig)	At farm gate
38	Crop-swine integrated system	At farm gate
39	Reduced dietary protein levels	At farm gate
40	Production in Catalonia	At farm gate
41	Production in Galicia	At farm gate
42	Supply chain management	At farm gate
43	Precision feeding	At farm gate
44	Production systems (organic)	At pork cutting gate
45	Production in Argentina	At farm gate
46	Individual data of performance and excretion	At retail gate
47	European local breeds	At farm gate
48	Altering genetic components of individual traits	At farm gate
49	Production system in Cuba	At farm gate
50	Dietary modification for fattening pigs	At farm gate
51	Feeding practices, animal health, and farm infrastructure	At farm gate
52	Production in Spain	At farm gate
53	Agroecosystems	At farm gate
54	Source of performance and excretion data	At farm gate
55	Housing conditions and manure management	At farm gate

**Figure 4:**
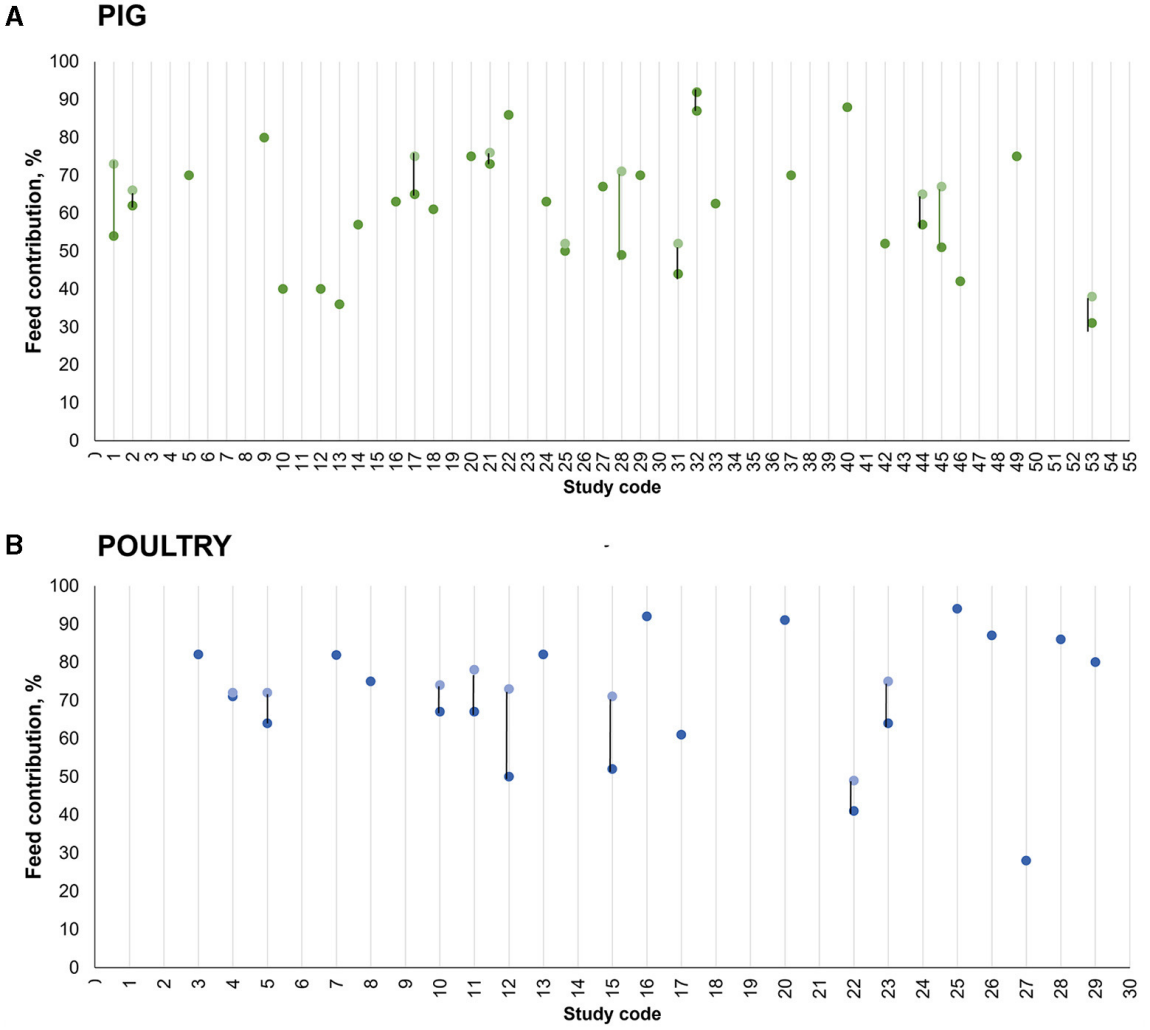
Feed contribution to the potential impact of climate change in LCA studies focusing on pig **(A)** or poultry **(B)** production. Study codes are the same as those presented in Tables 1, 2 for pigs and poultry, respectively. Blank lines were used for studies where the exact information was not presented in the original publication (text or tables, as the exact value could not be obtained when information was presented in figures).

Changes in feeding practices (diet composition or feeding programs) were studied in 25% of the papers. The relative participation of feed production (which includes each ingredient's life cycle, fabrication, and transport) varied from 31 to 76% of the overall greenhouse gas (GHG) emissions in the pig database (Figure 4A). Despite the importance of feeding to the total pig production impact, the diet composition used in the inventory was described by the minority of the papers. Only 38% of the papers described the ingredient formulas, while only 29% of the studies showed any description for dietary nutritional composition, limited sometimes to crude protein. In addition, the environmental impacts related to the production of individual ingredients were presented in only 9% of the papers. The proportion of total impact associated with feed was highlighted in most of the studies. However, the impact of feed production (considering as a functional unit; e.g., 1 ton of feed) was presented in only 15% of the publications. These data would be of great value for further investigations on feeding practices that may mitigate the potential environmental impact of pig production. In addition, more information on feeding practices would allow a better comparison among studies, as great variability exists between the final results (impact of pig production) presented by the studies even for the same functional unit.

As previously stated, crop production is a major contributor to the overall impacts of the pig production chain. The globalization of feed ingredient markets is relevant to LCA studies because it disconnects commodity production from its use/consumption. In a context in which most of the ingredients used for feed production are internationally traded, it is important to highlight that the impacts associated with a certain product are virtually shared with several countries involved in the international trade. The most frequent example of this intercontinental sharing was the use of soybean imported from South America, mainly from Brazil, in European countries. Considering the pig database, 49% of the studies mentioned the use of Brazilian soybean. For that reason, several papers also mentioned the inclusion of overseas transport during the inventory characterization.

### Studies Focusing on Poultry Production

The research process until obtaining the final poultry database is described in Figure 1B. Articles obtained by online searches (6,502 references) were critically evaluated, which resulted in several exclusions. Seventeen references were excluded when assessing the full-text (criterium i and ii: 2 publications; criterium iii: 1 publication; criterium iv: 7 publications; and criterium v: 7 publications). The final list of 30 selected studies is described in Table 3.

**Table 3:** Summary of the LCA studies on poultry production in terms of location, focus, functional unit and climate change potential.

**Code**	**Study**	**Country**	**Focus**	**Functional unit**	**Climate change potential^a^, CO_2_-eq**
1	Bennett et al. (59)	Argentina	Meat	1 kg (body weight) of broiler	NA
2	Mollenhorst et al. (60)	Netherlands	Egg	1 kg of eggs	3.9–4.6 kg
3	Pelletier (61)	United States	Meat	1 t (live weight) of broiler	1.40 t
4	Leinonen et al. (62)	United Kingdom	Meat	1 t of expected carcass	4.41–5.66 t
5	Leinonen et al. (63)	United Kingdom	Egg	1 t of marketable eggs	2.92–3.45 t
6	Leinonen et al. (64)	United Kingdom	Meat/Egg	1 t of expected carcass weight	3.54–4.39 t
7	Pelletier et al. (65)	United States	Egg	1 t of marketable eggs	2.95–3.46 t
8	Thévenot et al. (66)	Reunion Island (France)	Meat	1 t of produced eggs	4.20–6.10 t
9	González-García et al. (67)	Portugal	Meat	1 t of produced eggs	4.32–6.45 t
10	Leinonen et al. (68)	United Kingdom	Meat/Egg	1 t of liquid eggs	4.95–7.48 t
11	Prudêncio da Silva et al. (69)	Brazil, France	Meat	1 t of whole chickens packed	2.49 t
12	Taylor et al. (70)	United Kingdom	Egg	1 kg (live weight) of broiler	1.62 kg
13	Ghasempour and Ahmadi (71)	Iran	Egg	1 kg of chicken meat packed	2.46 kg
14	Kalhor et al. (72)	Iran	Meat	1 t of expected carcass weight	4.22–4.42 t
15	Kebreab et al. (31)	Europe, North, and South America	Meat	1 t of marketable eggs	2.83–2.92 t
16	Leinonen et al. (73)	United Kingdom	Meat	1 t (live weight) of broiler	1.45–2.70 t
17	Cesari et al. (74)	Italy	Meat	1 t of packaged chicken	1.95–4.02 t
18	Giannenas et al. (75)	Greece	Meat	1 dozen eggs	1.9–2.5 kg
19	Mainali et al. (76)	Bangladesh	Egg	1 kg of expected carcass	4.07 kg
20	Payandeh et al. (77)	Iran	Meat	1 t (live weight) of broiler	1.39–3.25 t
21	Pelletier (78)	Canada	Egg	1 t of packed meat	2.93–5.36 t
22	Pishgar-Komleh et al. (79)	Iran	Meat	1 t (live weight) of broiler	1.12–1.34 t
23	Wiedemann et al. (80)	Australia	Meat	1 kg (live weight) of broiler	3.03–3.84 kg
24	Abín et al. (81)	Spain	Egg	1 kg of carcass	5.52 kg
25	Skunca et al. (82)	Serbia	Meat	1 t of expected carcass weight	2.76 t
26	Arrieta and González (48)	Argentina	Meat	1 kg (live weight) of broiler	1.63–4.21 kg
27	Duarte da Silva Lima et al. (83)	Brazil	Meat	10,000 eggs	1.74 t
28	Ramedani et al. (84)	Iran	Meat	1 t (live weight) of broiler	5.00–5.78 t
29	van Hal et al. (85)	Netherlands	Egg	1 t of produced eggs	1.37–2.44 t
30	Estrada-González et al. (86)	Mexico	Egg	1,000 broilers	17.36–20.25 t

a *Original results were preserved, however, some conversions were needed for the purpose of having the same weight unit as the functional unit*.

The first study identified in the poultry database was published in 2006. Considering the entire database, 13 journals reported publications, with 10 papers being published in Journal of Cleaner Production and 5 papers in Poultry Science. Broiler production was evaluated in 18 studies, eggs were the main product evaluated in 10 studies, and both products were assessed in two papers. Production scenarios located in the United Kingdom and Iran (which were considered in 6 studies each); followed by Argentina, Brazil, France, Netherlands, and the USA, which were considered in two studies each; as illustrated in Figure 2B.

Likewise to the pig database, the scope described as cradle-to-farm gate was used in most studies focusing on poultry production. Impacts associated with slaughtering and processing were considered in 10 publications. Besides climate change, studies presented other impact categories (Figure 3B), such as acidification, eutrophication, and the use of energy and land. The characterization of the meat or egg production in the region or country was the main objective in 15 studies (Table 4).

**Table 4:** Summary of the LCA studies on poultry production in terms of main subject under analysis and scope boundary.

**Code**	**Main study subject**	**Focus**	**Scope final boundary**
1	Conventional and genetically modified maize	Meat	At processing plant door
2	Production systems	Egg	At farm gate
3	Production system in the United States	Meat	At farm gate
4	Production system in the United Kingdom	Meat	At farm gate
5	Production system in the United Kingdom	Egg	At farm gate
6	Alternative protein crops	Meat/Egg	At farm gate
7	Production system in the United States	Egg	At farm gate
8	Accounting for farm diversity	Meat	At farm gate
9	Production system in Portugal	Meat	At shell egg processor facilities
10	Welfare-enhancing system changes	Meat/Egg	At breaker facilities
11	Large and small-scale production in Brazil and France	Meat	At processor door
12	Production systems (free-range)	Egg	At farm gate
13	Production system in Iran	Egg	At processor door
14	Production system in Iran	Meat	At farm gate
15	Specialty feed ingredients	Meat	At farm gate
16	Genetic changes	Meat	At farm gate
17	Production system in Italy	Meat	At processor gate
18	Protease and replacement of soybean meal	Meat	At farm gate
19	Litter management	Egg	At farm gate
20	Mitigating environmental impacts by data envelopment analysis	Meat	At farm gate
21	Production system in Canada and housing systems	Egg	At processor door
22	Production system in Iran	Meat	At farm gate
23	Production system in Australia	Meat	At farm gate
24	Production system in Spain	Egg	At slaughterhouse gate
25	Chicken meat chain	Meat	At farm gate
26	Production system in Argentina	Meat	At farm gate
27	Production system in Brazil	Meat	At farm gate
28	Comparing ostrich and chicken production	Meat	At farm gate
29	Feed-food competition	Egg	At processor door
30	Production system in Mexico	Egg	At farm gate

Three papers described the environmental impacts of replacing ingredients in feed formulas, while one paper described the impacts of dietary supplementation with protease. Feeding was highlighted as the major source of environmental impact in most studies, accounting for 28–82% of the overall impact of climate change (Figure 4B). Despite the importance of feeding to the total impact, the diet composition used in the inventory was not described in most studies. Only 13% of the papers described the ingredient formulas, while only 10% of the studies showed any description for dietary nutritional composition. In addition, the environmental impacts related to the production of individual ingredients were presented in only 13% of the papers, with the impact of feed production (considering as a functional unit; e.g., 1 ton of feed) being presented in only 20% of the publications. The use of Brazilian soybean was reported by 30% of the studies, highlighting the importance of international trade also for the environmental impact of poultry production.

## Discussion

**Figure 5:**
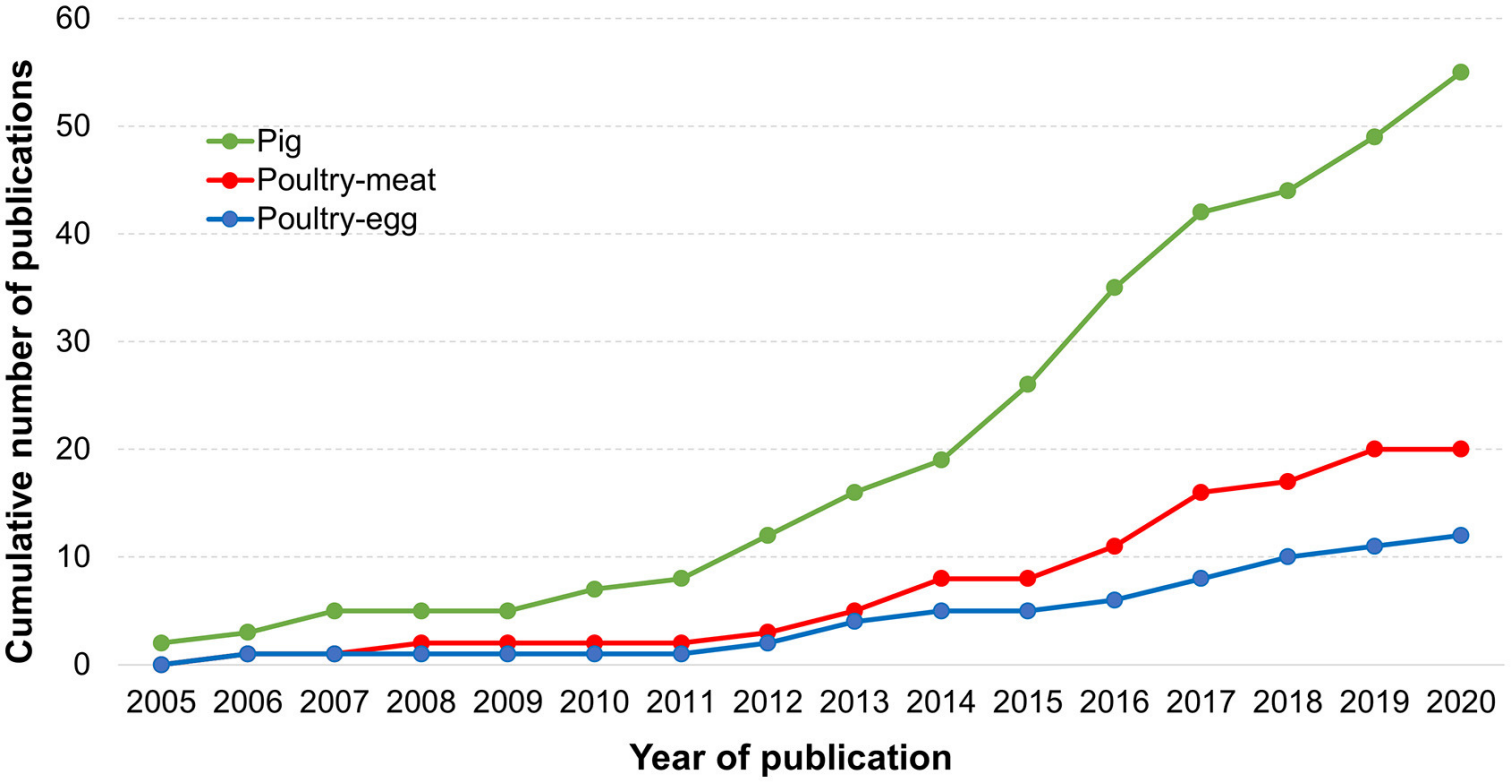
Cumulative number of LCA studies focusing on pig or poultry production.

The availability of peer-reviewed publications using LCA to assess the environmental impacts of pig and poultry production systems has increased over the years (Figure 5). The first studies of each database were published in close years for both pig (2005) and poultry (2006) production chains. However, the availability of studies focusing on pig production evolved greatly in the following years, mainly after 2014. In most research areas, the number of studies on poultry production is great than the number of publications available in a comparable topic in pigs. However, the opposite was found in this systematic review, probably due to the higher risk and concern with the environmental impacts of pig production compared to poultry systems.

The interest in using LCA to investigate the sustainability of a given production originates from its capability to quantify and evaluate the resources consumed and the emissions released at each phase needed for its production (8). Concerns about food safety and climate change have greatly increased in recent years. In response, the livestock industry must then reduce the utilization of resources by increasing its efficiency while reducing its environmental impact.

The impacts estimated for both production systems varied greatly across studies, mainly due to the heterogeneity of functional units and the amplitude of the considered life-cycle scopes. However, other attributes may also be listed as sources of variability when comparing publications. In particular, this heterogeneity may be related to the production systems under analysis (10, 62, 63), as well as to regional characteristics (31, 69). The conditions considered for housing (58), farm size (38, 79), level of intensification (20), and manure management (23) are also reported as important factors determining the final impact associated to the product. When focusing on animal aspects, some welfare (68) and genetic traits (50, 51, 73), as well as sanitary aspects (54) were reported.

Feed production was highlighted in several papers due to its relevant contribution to the total environmental impact. This phase was simulated including each ingredient's life cycle, fabrication, and transportation to the feed mill or to the farm in most studies. The reported contribution of the feed production phase relative to the overall GHG emissions varied from 31 to 76% in the pig database. In the poultry database, it accounted for 28–82% of the total climate change impact. Regardless of the exact environmental impact attributed to the feeding phase, almost all studies identified feeding as the production factor having the greatest environmental impact. These findings support the hypothesis that eco-friendly feeding practices can mitigate the environmental impacts of pig and poultry production.

### Importance of Rearing System Scenarios

Even though a comparison between organic and conventional systems will not be deeply reviewed, it is important to highlight that several studies indicated the production system as one of the important aspects determining the relative contribution of feeding to the overall environmental impact (due to the feed ingredient composition, number of feeding phases, among others). Conventional production systems were considered in most simulations (i.e., conventional feed ingredients). However, some studies evaluated the environmental impacts of adopting alternative production systems (e.g., organic, free-range, certified labels). According to Leinonen et al. (62, 63), the global warming impact necessary to obtain a given functional unit of feed (e.g., 1 ton) can be low in organic farms in comparison to conventional farms. However, a higher feed amount is generally necessary for organic farms than in conventional production systems to obtain the same functional unit. Several reasons are indicated in the papers, as the impairment in feed conversion ratio, an increase in feed consumption, or even waste of feed or products. Thus, when the total cycle is analyzed, a greater global warming potential impact may be associated with feeding animals in organic than in conventional systems (10, 62, 63).

The environmental impacts of a given rearing system are highly correlated with animal performance, especially feed efficiency (27, 87). Thus, technologies that improve animal performance usually have great potential to mitigate life cycle environmental impacts. In this particular aspect, some technologies were assessed in the reviewed studies. Immunological castration and feed additives are some of these factors (16, 31, 53, 75), but probably many more aspects still need to be evaluated in future research.

Another important factor evaluated in some studies was the impact of innovative practices during the cultivation or processing of feed ingredients. The use of maize genetically modified (59), different processing methods for soybean (48), or crop-animal integrated systems (41) were evaluated, and impacts in the final product (i.e., functional unit) were reported.

### Importance of Feeding Scenarios

The use of alternative feed ingredients is an important strategy in livestock systems. Some studies presented environmental advantages when using co-products in the assessed feeds (28, 33, 39). Other papers indicated that these advantages may be related to the calculation method, with favorable results being reported only when the impacts were not co-allocated between the main and the co-products (35, 88). The environmental cost to obtain co-products cannot be ignored in the LCA analysis, and this should be probably further evaluated in future research. Another limitation to be considered when comparing studies are the different ingredient choices given the difficulties in data acquisition, especially for local or limited ingredients as well as the great variability among processes used to obtain co-products (64, 88).

The distance between feedstuff production location and their place of use is an important argument in favor of using ingredient choice (or replacement) as a strategy to mitigate environmental impacts. Feed ingredients are products with cross-border flows, which are a consequence of globalization. Reducing the distance from producers to consumers means fewer transportation needs, and consequently fewer costs and emissions. Using this argument, several studies were developed proposing the use of locally grown ingredients instead of products cultivated in different countries or even continents (22, 43, 64). This is particularly important for local protein-ingredients that replace imported soybean and soybean meal (64). However, the use of local ingredients must not impair feed conversion (24, 66). Otherwise, the advantages may be lost caused by increased demand for feed to reach the same final weight.

Feed composition in terms of ingredients is also a way to reduce the excretion of nutrients and, consequently, manure composition. For that reason, the choice of ingredients needs to be made always with caution, focusing on the origin, but also on the nutritional quality of the product. Nitrogen excretion in manure is highly correlated with diet formulation. If an increase in nitrogen losses in the manure is related to a given ingredient choice, it is expected that this modification will lead to higher GHG emissions and probably other major consequences too (65). In this context, strategies that mitigate nutrient excretion, such as enzyme supplementation (75), synthetic amino acid partially replacing protein crops, or the use of low-protein diets (6, 42, 57), can potentially mitigate the environmental impacts of both pig and poultry production. The modification of the feed formulation method (89) and the adoption of precision feeding techniques (46, 90) are also very important and innovative tools. Due to its relevance for future animal production, precision feeding will be further discussed in the next section, with a focus on pig production.

### Precision Feeding as an Eco-Friendly Strategy to Mitigate the Environmental Impacts of Pig Production

Feeding is a major source of environmental impacts, as previously discussed. When correctly applied, precision feeding is an efficient tool to decrease production and environmental costs (91). Pigs and poultry are usually fed according to group requirements, disregarding individual particularities. This means that all animals receive the same feed for an extended period, with part of the population receiving nutrients above their requirements (92). The animals that receive nutrients above their needs excrete this excess. An increased protein intake decreases protein efficiency utilization, resulting in larger nitrogen excretions (93). In many pig commercial systems, the nitrogen retention in conventional phase-feeding programs will rarely exceed 35%, being that the efficiency of nitrogen utilization used in many LCA studies (94). However, nitrogen efficiency varies depending on age, sanitary status, and crude protein levels (95, 96).

Precision feeding consists in providing the right amount of feed with the right balance composition to each animal at the right time. Thus, precision feeding can be defined as the technology that provides each animal the nutrients tailored to meet in real-time the animal requirements (91). Nitrogen and phosphorus excretions can be decreased by 40% and consequently reduce production costs by 10% when using an individual precision feeding program (93, 97).

In this context, precision feeding can improve the sustainability of pig production systems. Automatic feeding stations allow pigs to be fed individually with a diet whose composition is appropriate to their growth potential (91). This strategy is an important pattern shift in animal nutrition because at this point nutritional requirements are no longer consider static, but as dynamic processes that develop differently for each individual.

The use of precision feeding instead of conventional group feeding systems already demonstrated several benefits. The increased nutrient-use efficiency and the consequent reduction in the excretion of polluting substances to the environment, improving the overall sustainability of the production system, are the main advantages presented by this feeding system (91). In addition, studies have shown that it is possible to considerably reduce soybean meal and dicalcium phosphate in diet formulations compared to conventional programs. In validation studies (93, 97), individual feeding allowed a reduction in lysine intake by up to 26%, and nitrogen and phosphorus excretion by 30 and 14%, respectively, without affecting the productive pig performance.

### Environmental Impacts of Applying Precision Feeding Techniques

Before applying precision feeding techniques, it is necessary to study the environmental impacts of adopting these techniques. An LCA study performed by Andretta et al. (46) intends to estimate the environmental impact of precision feeding techniques applied to pig production. Once again, in Brazilian scenarios, feeding was the largest source of environmental impact. In addition, the study showed that replacing conventional group feeding with daily group feeding (nutrient supply adjusted daily to meet the group requirements) could decrease the potential impact of eutrophication by 4% and acidification by 3%. The mitigation was even greater (up to 6% for the potential impact of climate change and 5% for eutrophication and acidification) when the program was applied to each animal individually (pigs received diets daily tailored to their requirements).

The study also highlighted a reduction over time in the potential impact of climate change associated with pig feed production related to reducing the expected dietary nutrient levels. In the simulated population, reducing the dietary standardized ileal digestible lysine level by one percentage point led to a reduction of up to 194.7 kg of CO_2_-eq per ton of feed, depending on the simulated scenario. Certainly, the main advantage of this method was the improved nutrient use efficiency. In other words, the same amount of product was produced using fewer resources. Monteiro et al. (34) performed a similar study considering Brazilian and French scenarios with simulated data (the previous study used data collected *in vivo*). In their study, a precision feeding system that fed pigs individually was able to reduce the impact of climate change by 7%.

### Future Challenges

Animals are exposed to several conditions during their lives and these factors may impact directly their nutrient requirements (98). For example, sanitary challenges affect the way amino acids are used by the animal because the nutrients that would be used for protein deposition are directed to cope with the immune system (98). Sanitary challenges also impact the growth performance of pigs and broilers (99), reducing feed efficiency and consequently increasing the environmental impact associated with this production (27). Cadero et al. (54) reported a significant effect of impaired health status on the carbon footprint of pig production. This is only one of several topics that need to be more evaluated in the future, especially in a scenario with reduced use of antibiotics in animal production.

Several studies on the environmental impact of animal production have been published, but only a few have worked using precision feeding programs or considering sanitary challenges. More studies must be carried out to better understand their environmental impact on modern pig and poultry production. Despite all the variability found in livestock, precision systems can foster some eco-friendly solutions by the possibility of managing animals as an individual, having their diets tailored based on real-time data.

### Important Aspects to Be Considered When Applying LCA to Animal Science

LCA is a well-known and established method to evaluate environmental impacts, particularly for complex production chains as those in the livestock sector. However, LCA has its limitation like any other scientific method. Some of these limitations have been described by Finkbeiner et al. (100). Some of these gaps may apply in the studies described in this systematic review.

One important limitation observed in the studies was the assessment of water use. Many studies did not include this impact category or they did not consider water consumption (water not returned to the system), which is very relevant for agriculture (100, 101).

The great variability in functional units is certainly another important limitation to be highlighted. The unit choice is a challenging task because it impacts directly on the results and is also related to the objective and scope (100). However, the variability among studies is a great limitation when comparing results since transformations are sometimes not possible or precise (e.g., results expressed for 1 ton of live pig are difficult to compare to those expressed for 1 ton of carcass because there are more processes included and sometimes the carcass yield is not fully known).

In addition, impacts on human health are probably insufficiently covered in LCA studies dealing with pig and poultry production. Soil contamination, noise, and odors are some of these impacts that are not commonly addressed in LCA studies. Additionally, the LCA method fails to consider other aspects, such as biodiversity, welfare, and social aspects (100). The positive impact of specific activities may be also disregarded.

Finally, the choice of a single scenario to represent the reality of an entire production chain is another important limitation of some reviewed LCA studies. The issue related to data gathering was previously highlighted (102). A single model (e.g., data collected in a single scenario) are not able to describe the pig and poultry production systems worldwide, and neither probably across regions. Even in integrated systems that are characterized by a higher level of uniformity, it is possible to observe a different performance in each producer (for the same genetic type, with the same feed, and similar management practices). Thus, variability is something that needs to be considered in future LCA studies.

## Conclusion

This systematic review confirmed feeding as the largest source of environmental impact associated with pig and poultry production systems. This supports the hypothesis that novel feeding techniques may mitigate the environmental footprint associated with both production chains. Precision feeding is highlighted as a way to optimize nutrient-use efficiency and, for that reason, as a promising tool toward more sustainable animal production systems. It is still a challenging task to properly consider and compare the variability among LCA studies. Despite these issues, LCA is a comprehensive way to assess sustainability from a global perspective and its application on pig and poultry production systems is very encouraged in future research.

## Data Availability Statement

The raw data supporting the conclusions of this article will be made available by the authors upon request.

## Author Contributions

AR, CF, CO, MK, and IA searched articles for the systematic review and interpreted results. IA interpreted results, prepared figures, and tables, and wrote the first draft of the manuscript. M-PL-M and CP were involved in the interpretation and discussion of results. FH and AM contributed to manuscript revision, read, and drafted the final version of the manuscript. All authors read and approved the final version of the manuscript.

## Conflict of Interest

The authors declare that the research was conducted in the absence of any commercial or financial relationships that could be construed as a potential conflict of interest.

## Publisher's Note

All claims expressed in this article are solely those of the authors and do not necessarily represent those of their affiliated organizations, or those of the publisher, the editors and the reviewers. Any product that may be evaluated in this article, or claim that may be made by its manufacturer, is not guaranteed or endorsed by the publisher.
